# Ice Houses

**Published:** 1879-05

**Authors:** 


### ICE-HOUSES.

THE *American Agriculturist* says: "First, the ground selected must be dry, and out of the way of floods, if near a stream; for if water stands in contact with the ice, it will melt away almost 'like the morning cloud.' It is well to have the ice-house on the north side of a hill, or of a house or big tree. If close to the house, and a cool-room can be made between it and the house, that will be found very convenient, and the ice-house wall next the cool-room need not be made so thick as on the other sides; in fact, a double boarding, with an inch of space between, is enough. It is well to dig out the ground so as to set the house a little lower than the general level, and it may be several feet lower if convenient. The bottom ought to slope to the middle or to one side, and to be grouted; that is, laid with broken stones which are covered with hydraulic cement mortar, poured over and in among them, and smoothed off even on the surface. The inclination of the bottom should lead to a sealed drain, so protected that it can not be stopped up by accident, or by sawdust. It is important that the drainage of an ice-house, whether the bottom be cemented as we have described or not, should be perfect, and that a circulation of air should not take place through the drain. This is easily affected by having the end of the drain (a round tile) rise two or three inches in a cemented depression, or basin, and turning over it a common flower-pot with the hole stopped.

"A house 10 × 10, or 12 × 12 feet, and eight feet from the bottom to the eaves, with a half-pitch roof, is about what is wanted on an ordinary farm, and will hold and keep more ice than is usually needed. The sides should be ten inches thick, the frame being of eight-inch uprights, of two-inch plank, set four on a side, (the end ones being a foot from the outside corners,) upon sills of the same width. The inside boarding should be of cheap inch stuff. The outside may be clapboarded, or boarded up and down and battened. Dry sawdust, planing-mill shavings, or dry spent tan-bark, may be used to fill in between the outer and inner boarding, and the filling should be settled down solid. The plates may be of two-inch plank; the rafters four on each side, of two-inch plank, six inches wide. They should be boarded outside and inside, and the space filled with shavings. The roof should be thatched or shingled, and the gable ends double boarded and filled like the sides. The door should be in one of the ends, four to six feet from the ground, and four feet high; and close to the peak there should be a sliding shutter for a ventilator. There should be a flooring not nailed down but laid firmly, to support the ice.

"The sides may rest on the grouting, or on a stone underpinning. When they are laid, they should have a coat of coal-tar all over, and when the house is done, sawdust stirred up with coal-tar should be filled into all the crevices and holes near the ground outside and inside, and earth heaped up around the sides and trodden down. Paint the sides with tar as high as the earth comes. How to fill an ice-house will be a subject for our December number.

"Straw Ice-Houses.—Where there is a great abundance of straw, ice may be preserved throughout the year, if packed in a compact mass and well covered with straw, perfect drainage being secured."

